# Identification of copy number variations among fetuses with ultrasound soft markers using next-generation sequencing

**DOI:** 10.1038/s41598-018-26555-6

**Published:** 2018-05-25

**Authors:** Jing Wang, Lin Chen, Cong Zhou, Li Wang, Hanbing Xie, Yuanyuan Xiao, Daishu Yin, Yang Zeng, Feng Tang, Yunyuan Yang, Hongmei Zhu, Xinlian Chen, Qian Zhu, Zhiying Liu, Hongqian Liu

**Affiliations:** 1 Department of Obstetrics and Gynecology, West China Second University Hospital, Sichuan University, Chengdu, 610041 China; 2 Key Laboratory of Birth Defects and Related Diseases of Women and Children, Ministry of Education, Sichuan University, Chengdu, 610041 China

## Abstract

A prospective analysis investigating the associations between pathogenic copy number variations (pCNVs) and ultrasound soft markers (USMs) in fetuses and evaluating the clinical value of copy number variation sequencing (CNV-seq) in such pregnancy studies was carried out. 3,398 unrelated Chinese women with singleton pregnancies and undergone amniocentesis at 18–36 weeks of gestation for fetal CNV-seq were included. According to the prenatal fetal ultrasound screening results, the samples were divided into 3 groups: normal ultrasound (n = 2616), solitary USM (n = 663), and two or more USMs (n = 119). CNV-seq was performed successfully using all samples. The prevalence of pCNVs in fetuses with normal ultrasound and USMs was 3.03% (79/2616) and 2.94% (23/782), respectively. The risk of segmental aneuploidies was significantly higher in the two or more USMs group (5/119, 4.20%) than in the normal ultrasound (27/2616, 1.04%) or solitary USM (9/663, 1.36%) groups (p = 0.002 and p = 0.031, respectively). Assuming that the resolution of karyotyping is ~5 Mb, a cytogenetic analysis would miss 33 of 102 (32.35%) pCNVs in these samples. Our results suggest an association between pCNVs and fetal USMs; multiple USMs indicate an increased risk of fetal segmental aneuploidies. In prenatal diagnostic testing, CNV-Seq identified additional, clinically significant cytogenetic information.

## Introduction

Ultrasound has become a crucial tool in the field of fetal medicine. In particular, first and second trimester scans have become a routine part of antenatal care to detect congenital anomalies that are solitary or part of an underlying chromosomal anomaly. Minor sonographic features of fetal aneuploidy, most commonly Down Syndrome (DS), were first reported in the 1980s^1^. As ultrasound technology has advanced, the detection of these soft markers has become easier. A large number of other ultrasound findings have been reported to be associated with common trisomy 21, 18, and 13, including nuchal fold thickening, ventriculomegaly, short femur or humerus, hypoplastic nose, echogenic bowel, pyelectasis, sandal gap toes, choroid plexus cysts, enlarged cisterna magna, single umbilical artery, and echogenic intracardiac foci^2,3^. Some soft markers have no significance beyond the association with aneuploidy, while some have potential implications that require evaluation and follow-up^4,5^. However, Some scholars have recommended that, when an abnormality/marker is detected at routine ultrasound examination, It is best to base counseling on an individual estimated risk for a chromosomal defect, rather than the arbitrary advice that invasive testing is recommended because the risk is high^6^. Therefore, the increased detection of ultrasound soft markers (USMs) can lead to confusion regarding the appropriate parental counseling and fetal management. Even if the pregnant woman has undergone amniocentesis or chorionic villus sampling (CVS) because of the presence of a USM in the fetus, the most common follow-up method reported in the literatures was karyotyping, which does not routinely detect small genomic deletions and duplications.

Copy number variations (CNVs) indicate regions of the DNA sequence with gains or losses and occur at high frequency in the human genome; these CNVs can affect the whole or segmental genome^7^. CNVs can be either benign or pathogenic, depending on their location and genetic content. Pathogenic CNVs (pCNVs) are increasingly associated with complicated diseases, especially with some structural congenital anomalies^8^. A recent study suggests an association between pCNVs and fetal isolated mild ventriculomegaly; pCNVs may also be involved in the pathological process of fetal isolated mild ventriculomegaly and postnatal neurodevelopmental disorders^9^. However, limited data exist on whether the presence of other USMs indicates an increased risk of pCNVs, except for common trisomy 21, 18, and 13. The potential association between pCNVs and fetal USMs is unclear, especially as this association relates to segmental aneuploidies.

The fetal sonographic anatomical screening is usually performed at 22–25 weeks of gestation in China, because many cardiac defects are not detected until relatively late in the second trimester of pregnancy^10^. Therefore, most USMs are detected at 22–25 weeks of gestation. If these pregnant women choose to diagnose fetal chromosomal abnormalities through prenatal diagnosis, conventional cytogenetic analysis most commonly involves cell culture from the amniotic fluid, but fetal fibroblasts obtained after 24 weeks of gestation take longer to grow (often longer than 2 weeks) with a higher culture failure rate than those sampled earlier in gestation^11^. Fetal blood sampling by puncture of the umbilical cord vein provides an alternative, quicker method for karyotyping in late pregnancy, but it is technically more difficult and has a higher miscarriage rate. Many prenatal services have developed multiplex fluorescence *in situ* hybridization (FISH), quantitative fluorescence polymerase chain reaction (QF-PCR), bacterial artificial chromosome on beads (BOBs), or multiplex ligation-dependent probe amplification (MLPA) for amniocenteses collected after 24 weeks, but these approaches are limited by the number of target chromosomes tested per assay.

Array comparative genomic hybridization (aCGH), first proposed in 1997^12^, has served as a robust and effective approach to screen for CNVs^13^. However, aCGH is expensive and has limited resolution and accuracy. Today, rapidly developing next generation sequencing (NGS) technologies provide a sensitive and accurate alternative approach to assess genomic variations. The quality, speed, and affordability give NGS a significant advantage over microarrays^14–16^. Thus far, there is little data reporting the use of NGS copy number variation sequencing (CNV-seq) for prenatal karyotyping in fetuses with USMs. In this prospective study, we analyze the frequency of pCNVs in fetuses with USMs and evaluate the application of CNV-seq as a tool for the identification of pCNVs.

## Results

Prospectively, we studied 3,398 unrelated Chinese women with a singleton pregnancy who were referred for an amniocentesis procedure to investigate the presence of a potential chromosome abnormality in the fetus. Based on the prenatal fetal ultrasound screening results, the samples were divided into 3 groups: normal ultrasound (n = 2616), solitary USM (n = 663), or two or more USMs (n = 119) (Table 1). CNV-seq was performed for all samples successfully. Overall, CNV-Seq revealed that 3,296 samples were euploid (97.00%) and 102 samples contained pCNVs (3.00%). Of these 102 cases with pCNVs, 33 (32.35%) had submicroscopic CNVs (smaller than 5 Mb).

**Table 1:** Pathogenic copy number variations (pCNVs) in different fetal risk groups.

Group		Sample no.	Euploid no.(%)	pCNVs				
				no.(%)	Whole Chromosome Aneuploidies no.(%)	Segmental Aneuploidies		
						no.(%)	≥5 Mb no.(%)	<5 Mb no.(%)
Normal Ultrasound		2616	2537 (96.97%)	79 (3.03%)	52 (1.99%)	27 (1.04%)^A^	2 (0.08%)	25 (0.96%)
USMs		782	759 (97.06%)	23 (2.94%)	9 (1.15%)	14 (1.79%)	6 (0.77%)	8 (1.02%)
Solitary USM		663	646 (97.44%)	17 (2.56%)	8 (1.21%)	9 (1.36%)^B^	3 (0.45%)	6 (0.91%)
2 or more USMs		119	113 (94.96%)	6 (5.04%)	1 (0.84%)	5 (4.20%)^C^	3 (2.52%)	2 (1.68%)
Total no. (%)		3398	3296 (97.00%)	102 (3.00%)	61 (1.80%)	41 (1.21%)	8 (0.24%)	33 (0.97%)

Chi-squared (X2) test was applied to compare pCNVs detection rate in different groups, A vs C: p = 0.002, B vs C: p = 0.031.

The mean gestational age and maternal age at the time of amniocentesis for the group with normal ultrasound results (n = 2,616) were 21.8 weeks (range 18.2–35.6) and 32.0 years (range 15.0–46.0), respectively. In this group, a total of 79 pCNVs (3.03%) were detected; of these, 27 (34.18%) had segmental aneuploidies. Additionally, 782 fetuses presenting with USMs (either solitary USM or multiples USMs) were included in this study. The mean gestational age and maternal age at the time of amniocentesis were 26.3 weeks (range 18.5–36.0) and 28.2 years (range 17.0–45.0), respectively. The most common USM was echogenic focus in the heart (486 samples, 62.15%), followed by mild ventriculomegaly (67 samples, 8.57%) and mild pyelectasis (62 samples, 7.93%). A total of 23 pCNVs (2.94%) were detected from these 782 fetuses. In samples from the 663 fetuses with a solitary USM, 17 (2.56%) featured pCNVs. Of these, 9 (52.94%) were segmental aneuploidies. Out of the 119 fetuses with two or more USMs, 6 pCNVs (5.04%) were detected, and 5 were segmental aneuploidies.

The risk of segmental aneuploidies was significantly increased in the group with two or more USMs (5/119, 4.20%) when compared with the group with normal ultrasound (27/2616, 1.04%) or a solitary USM (9/663, 1.36%) (p = 0.002 and p = 0.031, respectively). Out of the 782 fetuses with USMs, 9 whole chromosome aneuploidies were found in 9 cases (1.15%); 4 were DS, 2 were T18, and the remaining 3 featured different types of sex chromosome aneuploidies. These whole chromosome aneuploidies were all confirmed by QF-PCR or FISH (Table 2). Also, there were 14 cases (1.79%) with segmental aneuploidies; of these, 10 cases (10/14, 71.43%) featured microdeletions or microduplications associated with known chromosome disease syndromes. All segmental aneuploidies were confirmed by karyotyping or aCGH (Table 3). Most patients (8/14, 57.14%) with a fetus with segmental aneuploidies consented to parental gDNA studies to complete a full trio analysis. It was found that 5 of the segmental aneuploidies were formed de novo (5/8, 62.5%) and 3 were inherited (3/8, 37.5%).

**Table 2:** Whole chromosome aneuploidies of the 782 fetuses with USMs.

Sample Number	USMs	Gestational Age (weeks)	Maternal Age (years)	Whole Chromosome Aneuploidies	Confirmation Test	Follow-up Outcome
P16	single umbilical artery	22	28	trisomy 18	QF-PCR	termination of pregnancy
P21	choroid plexus cyst	20.3	27	trisomy 18	QF-PCR	termination of pregnancy
P12	thickened nuchal fold	20.6	42	trisomy 21	QF-PCR	termination of pregnancy
P14	thickened nuchal fold	19.6	29	trisomy 21	QF-PCR	termination of pregnancy
P15	absence of nasal bone	26	29	trisomy 21	QF-PCR	termination of pregnancy
P23	echogenic focus in the heart	27.2	29	trisomy 21	QF-PCR	termination of pregnancy
P22	echogenic focus in the heart, tricuspid regurgitation	24	27	47, XXY	QF-PCR	normal development at 3 months
P19	choroid plexus cyst	32.5	31	47, XYY	QF-PCR	normal development at 6 months
P13	thickened nuchal fold	20.2	35	48, XXXX	FISH	termination of pregnancy

**Table 3:** Segmental aneuploidies of the 782 fetuses with USMs.

Sample Number	USMs	Gestational Age (weeks)	Maternal Age (years)	Segmental Aneuploidies (size) (hg19)	Known Chromosome Disease Syndromes	Origin	Confirmation Test	Follow-up Outcome
P17	echogenic focus in the heart, persistent left superior vena cava	23	30	del1q21.1q21.2 (1.26 Mb)	1q21.1 recurrent microdeletion	unknown	CMA	termination of pregnancy
P5	tricuspid regurgitation	29.3	29	del1q21.1q21.2 (1.34 Mb)	1q21.1 recurrent microdeletion	maternal	CMA	termination of pregnancy
P2	thickened nuchal fold	21.2	30	del1q21.1 (0.36 Mb)	1q21.1 susceptibility locus for Thrombocytopenia-Absent Radius syndrome	unknown	CMA	termination of pregnancy
P8	mild ventriculomegaly, mild pyelectasis	22.5	26	dup3q26.33q29 (15.28 Mb) del10q26.3 (2.54 Mb)	3q29 microduplication syndrome	Maternal balanced reciprocal translocation	CMA	termination of pregnancy
P7	mild ventriculomegaly	33.3	39	del5p15.33p13.3(33.38 Mb)	Cri du Chat Syndrome	unknown	CMA	termination of pregnancy
P3	thickened nuchal fold	20.3	27	del17q12 (1.36 Mb)	renal cysts and diabetes	de novo	CMA	termination of pregnancy
P6	mild pyelectasis	23.6	26	del17q12(1.46 Mb)	renal cysts and diabetes	unknown	CMA	termination of pregnancy
P9	dilatation of the umbilical vein, short long bones	22	39	del7q11.23 (3.80 Mb)	Williams-Beuren Syndrome	de novo	CMA	termination of pregnancy
P4	Absence or dysplasia of nasal bone	27.1	30	del4p16.3p15.1 (29.50 Mb)	Wolf-Hirschhorn Syndrome	unknown	CMA	termination of pregnancy
P1	echogenic focus in the heart	26.5	25	del16p13.11 (1.18 Mb)	16p13.11 recurrent microdeletion	de novo	CMA	no structural abnormality visible until 37 weeks of gestation
P10	echogenic focus in the heart	24	26	del3p26.3p26.2 (2.60 Mb)	—	de novo	CMA	normal development at 4 months
P11	short long bones	28.3	27	dup11q14.1q25 (57.24 Mb) delXp22.33p11.22 (50.76 Mb)	—	unknown	karyotyping	termination of pregnancy
P18	single umbilical artery, short long bones, tricuspid regurgitation	25.2	40	dup8p23.1p21.2 (12.92 Mb) del8p23.3p23.1 (6.78 Mb)	—	de novo	CMA	termination of pregnancy
P20	mild ventriculomegaly, mild mitral and tricuspid regurgitation	22.4	37	dup1q32.2q44 (41.80 Mb) dup15q11.1q13.2 (10.20 Mb)	—	Maternal balanced reciprocal translocation	karyotyping	termination of pregnancy

## Discussion

Chromosomal abnormalities are a serious threat to human health; some fetuses with chromosomal abnormalities have obvious structural abnormalities by ultrasound, but some do not. USMs are often found in fetuses with whole chromosome aneuploidy (mostly in DS) and congenital structural anomalies. However, USMs can also be found as normal variants in a substantial proportion of euploid fetuses without structural anomalies. USMs are detected during fetal sonographic anatomical screening in approximately 10% of normal fetuses in the USA^17^. Ginsberg showed that there is a high recurrence rate of solitary USMs, which supports the hypothesis that genetic factors form the basis for the reappearance of USMs^18^. Therefore, there were different incidences of USMs among fetuses of different races.

In our study of a population of 3,398 singleton pregnancies, USMs were detected in 23.01% of the fetuses. CNV-Seq detected 9 whole chromosome aneuploidies from the 782 fetuses with USMs, and DS was the most common abnormality. These results coincide with those reported previously in the literatures^1,19^. The incidence of pCNVs in fetuses with USMs was 2.94% (23/782), which is similar to that in fetuses with a normal ultrasound (79/2616, 3.03%). The group with normal ultrasound results featured patients with advanced maternal age or a high risk for chromosomal abnormality based on maternal serum screening; these are high risk factors for whole chromosome aneuploidies^20^. The estimated prevalence of chromosomal abnormalities is 0.1–0.65% for newborns^21,22^. Our findings show that the presence of USMs is associated with an increased risk of pCNVs compared to the risk in the general pregnant population.

In the 782 samples with USMs in our study, whole chromosome aneuploidies did not increase as the number of USMs increased. The frequencies of segmental aneuploidies in the normal ultrasound, solitary USM, and two or more USMs groups were 1.04%, 1.36%, and 4.20%, respectively. The risk of segmental aneuploidies in the two or more USMs group was significantly increased compared to the risk in the other groups. Accordingly, our results suggest that there is an association between segmental aneuploidies and multiple USMs, which may be a useful prenatal clinical indicator for segmental aneuploidies.

Based on the assumption that the resolution of karyotyping is on the order of 5 Mb, a cytogenetic analysis of these 3,398 samples would have detected all 61 whole chromosome aneuploidies, but missed 33 of the 41 pathogenic segmental aneuploidies, giving a detection rate of 2.03% (69/3398). In contrast, the detection rate using CNV-Seq was 3.00% (102/3398), potentially increasing the detection of pCNVs by 0.97% when compared to karyotyping. Submicroscopic CNVs are an important cause of developmental delay, intellectual disability, and autism spectrum disorders in children^23^. In the context of prenatal diagnostic testing, high resolution detection of chromosome abnormalities identified additional, clinically significant cytogenetic information that was missed with karyotyping^24^.

**Figure 1:**
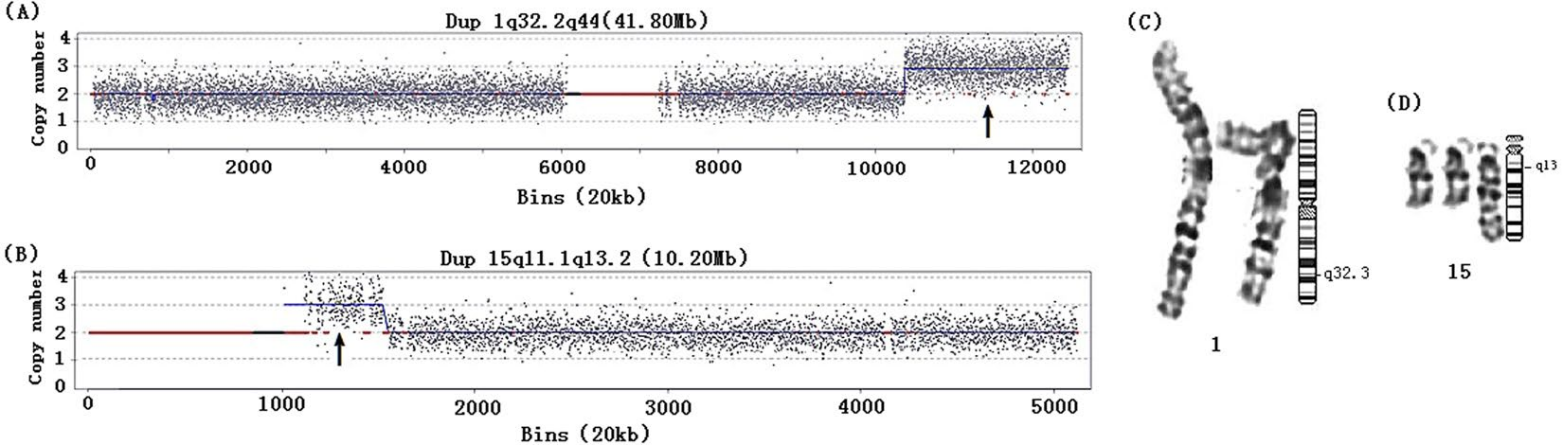
The CNV-seq profiles and partial karyograms of the fetus. (**A**,**B**) CNV-seq profiles for dup1q32.2q44 and dup15q11.1q13.2 (arrows), data is plotted as copy number (Y-axis) versus 20 kb chromosomal read bins (X-axis). The mean copy number along the length of each chromosome is indicated by the blue line. (**C**,**D**) The amniocytes of P20 showed partial karyotype and ideogram with an additional derivative chromosome der (15) t(1; 15) (q32.2; q13).

At the time of this study, 4 of the 23 fetuses with pCNVs detected with USMs had not been terminated; 3 fetuses have been born and examined by a pediatrician to reveal no obvious features of a disease syndrome. However, because the children are very young and the clinical phenotype may not have appeared yet, a follow-up is necessary for these children. In the 14 fetuses with segmental aneuploidies, obvious structural abnormalities were detected in 3 fetuses by subsequent ultrasound: 1 case (P3) featured bilateral renal enlargement with echo enhancement, 1 case featured bilateral small kidneys (P4), and 1 case (P20) featured a ventricular septal defect, pulmonary stenosis, and polyhydramnios. The amniocytes from P20 were 47, XX, t(1; 15) (q32.2; q13), +der (15) t(1; 15) (q32.2; q13)mat, whereas the karyotype of the mother was 46, XX, t(1; 15) (q32.2; q13), and the father showed the normal karyotype 46, XY. The couple already had a 12-year old daughter with a normal phenotype who had not yet undergone a chromosome analysis. The CNV-seq profiles and partial karyograms of the fetus are shown in Fig. 1. Additionally, a fetus (P8) with dup3q26.33q29 (15.28 Mb) and del10q26.3 (2.54 Mb) because of a maternal balanced reciprocal translocation was detected. Finally, P5 featured a 1q21.1 recurrent microdeletion, which was inherited from the mother, whose only phenotype was irritability.

USMs of aneuploidy were originally proposed to assess the risk for DS in women of advanced maternal age. Current ultrasound and serum screening protocols have far better sensitivity and specificity than age alone^25^, and screening by the analysis of cell-free DNA in the maternal blood in singleton pregnancies can detect >99% of fetuses with DS, 98% with T18, and 99% with T13^26^. When the aneuploidy risk is no longer a concern, follow-up for such patients should include consideration of other non-aneuploidy-related associations, such as chromosomal structural abnormalities and congenital structural anomalies.

In conclusion, our results suggest that there is an association between pCNVs and fetus USMs; multiple USMs are associated with an increased risk of fetal segmental aneuploidies. Identifying specific genomic alterations may provide insight into the pathogenetic mechanism and aid in better diagnosis and prognosis for adverse outcomes from fetal USMs. The accuracy of pCNV detection by CNV-Seq in 102 patients was also assessed using a range of other gold-standard molecular techniques. All confirmatory results from the validation tests were consistent with the original CNV-Seq diagnoses, indicating a 100% concordance rate. In the context of prenatal diagnostic testing, CNV-Seq identified additional, clinically significant cytogenetic information that was missed with karyotyping.

## Materials and Methods

### Biological Samples

This study involved pregnant women who underwent amniocentesis at 18–36 weeks of gestation for fetal CNV-seq at the West China Second University Hospital of Sichuan University between February and September 2017. None of the fetuses showed any other structural abnormalities by ultrasonogram before amniocentesis. Samples were categorized into 3 different fetal risk groups: fetuses with normal ultrasound, solitary USM, or two or more USMs. The fetal group with normal ultrasound consisted of patients with advanced maternal age (AMA, over 35 years at the expected date of childbirth) and high risk for DS or trisomy 18 (T18) based on maternal serum screening. USMs included thickened nuchal fold, echogenic bowel, mild ventriculomegaly (range 10–15 mm)^27^, echogenic focus in the heart, choroid plexus cyst, enlarged cisterna magna, mild pyelectasis (dilatation of the renal pelvis ≥4 mm in the 2nd trimester and ≥7 mm thereafter), absence or dysplasia of nasal bone, short long bones, tricuspid regurgitation, persistent left superior vena cava, and umbilical cord abnormality (single umbilical artery or dilatation of the umbilical vein). The diagnosis of USMs was confirmed by a second experienced ultrasonographer. As a quality control measure for the amniocyte DNA, maternal DNA contamination from the procedure was assessed by QF-PCR using a set of 15 highly polymorphic short tandem repeat (STR) markers in all samples. If the sample was deemed to have maternal DNA contamination, the amniocyte DNA sample was considered unacceptable for a reliable chromosome test result. The other amniotic fluid samples from the patient were used for cell culture following the standard protocols^28^, and the CNVs were analyzed from the genomic DNA (gDNA) isolated from the cultured amniotic fluid cells. The study was approved by the Institutional Ethics Committee of Sichuan University, and test results were used for research with the informed consent of the parents. We confirm that all methods were performed in accordance with the relevant guidelines and regulations.

### Copy Number Variation Sequencing (CNV-seq)

RNA-free high-molecular-weight DNA was isolated from amniotic fluid using the DNeasy Blood and Tissue Kit (Qiagen GmbH, Hilden, Germany). The quality and concentration of gDNA from the samples was assessed using the Qubit 2.0 Fluorometer (Thermo Fisher Scientific, Waltham, Massachusetts, USA). gDNA (50 ng) was fragmented, and DNA libraries were constructed by end filling, adapter ligation, and PCR amplification. DNA libraries were subjected to massively parallel sequencing using the NextSeq CN500 platform (Illumina) to generate 5 million raw sequencing reads with 36-bp genomic DNA sequences. Using the hg19 genomic sequence as a reference, a total of 2.8–3.2 million reads were uniquely and precisely mapped using the Burrows Wheelers algorithm^29^. Mapped reads were allocated progressively to 20 kb bin sizes from the p to q arms of the 24 chromosomes. Counts in each bin were then compared between all test samples run in the same flow cell to evaluate copy number changes using previously described algorithms^30,31^. Chromosome profiles were finally plotted as copy number (Y-axis) versus 20 kb count window (X-axis). A blue line was used to indicate the mean copy number across each chromosome to identify the nature and map position of any deleted or duplicated regions. Identified and mapped CNVs were interrogated using publicly available databases, including DECIPHER (https://decipher.sanger.ac.uk/), DGV (http://dgv.tcag.ca/), 1000 genomes (http://www.internationalgenome.org/), OMIM (http://omim.org/), and PubMed (https://www.ncbi.nlm.nih.gov/pubmed). CNVs were classified into three categories: benign CNVs, variants of uncertain significance (VOUS), and pCNVs. CNVs coinciding with known polymorphic CNVs were interpreted as benign. CNVs were defined as pathogenic if they were identified in regions of known clinical relevance or in genes associated with known disease phenotypes. CNVs that did not fit any of the above criteria were categorized as VOUS. In this study, we only focused on pCNVs. Additionally, we examined parental gDNA (if available) to determine if the pCNVs identified were formed de novo or inherited.

### Confirmatory testing of pCNVs

QF-PCR for chromosomes 13, 18, 21, X, and Y was performed for all samples. The STR markers used were D21S1435, D21S1411, and D21S11 for chromosome 21; D18S1002, D18S391, D18S535, and D18S386 for chromosome 18; D13S628, D13S742, D13S634, and D13S305 for chromosome 13; and DXS981, DXS6809, DXS22, and AMXY for the X and Y chromosomes. PCR was performed using a Bio-Rad PTC-200 PCR system (Bio-Rad, Mexico City, Mexico). The PCR products were analyzed using an ABI 3500 automated sequencer (Applied Biosystems, Tokyo, Japan). Sizing and labeling alleles were collected with Gene Mapper software (Applied Biosystems, Foster City, CA, USA). Allele dosage ratios between 0.8 and 1.4 were defined as normal, while an allele ratio >1.8, <0.65, or the presence of three alleles of equal area indicated trisomy. A single peak was described as uninformative^32^. When all markers for a chromosome were uninformative or the QF-PCR failed to have a result, enumeration was done by FISH (GP Medical, Beijing, China). Amniocytes were assessed for aneuploidy according to the manufacturer’s protocol (http://www.gpmedical.com.cn) as previously described^33^. Amniotic fluid samples were used for cell culture following the standard protocols^28^. Cells were cultivated with AmnioMAX-II (Gibco, Carlsbad, Calif., USA) medium. Chromosome preparations were G-banded using trypsin-Giemsa for karyotyping. A minimum of ten metaphases at the 400–500 band resolution level were routinely analyzed for each specimen. Chromosome karyotype map scanning and acquisition were done using an automatic metaphase chromosome analysis system (MetaSystems, Göttingen, Germany). Karyotypes were analyzed by two qualified senior laboratory technicians. aCGH was performed using the CGX v2 Oligo aCGH Kit (manufactured by Agilent Technologies, Santa Clara, CA, USA; Perkin Elmer, Turku, Finland). The microarray was scanned using the Agilent SureScan Microarray Scanner (Agilent, Santa Clara, CA, USA). Data were extracted using the Agilent CytoGenomics software (Agilent, Santa Clara, CA, USA) and analyzed using the Genoglyphix Analysis software (Perkin Elmer, Waltham, MA, USA).
